# Ferroptosis-related gene signatures in neuroblastoma associated with prognosis

**DOI:** 10.3389/fcell.2022.871512

**Published:** 2022-09-06

**Authors:** Yiru Chen, Zihao Li, Qingtai Cao, Haoyu Guan, Longfei Mao, Mingyi Zhao

**Affiliations:** ^1^ Department of Pediatrics, The Third Xiangya Hospital, Central South University, Changsha, China; ^2^ Xiangya School of Medicine, Central South University, Changsha, China; ^3^ Department of Pharmacy, College of Biology, Hunan University, Changsha, China; ^4^ Transplantation Center, The Third Xiangya Hospital, Central South University, Changsha, China

**Keywords:** neuroblastoma, ferroptosis, prognosis, stage 1, stage 4

## Abstract

**Background:** Ferroptosis, a form of regulatory cell death, has been linked to the development of various tumors. Peripheral neuroblastoma (NB) is one of the most common extracranial solid tumors in children, and it has been proposed that regulating tumor cell ferroptosis may be a future treatment for NB. However, it is unclear how ferroptosis contributes to NB development.

**Methods:** Expression data were collected from two independent cohorts (GEO and Arrayexpress databases). Univariate Cox analysis, multivariate Cox analysis, and the least absolute shrinkage and selection operator (Lasso) algorithm were applied to create a prognostic signature, whose performance was quantified using the area under the receiver operating characteristic curve (AUC) and Kaplan–Meier curves. A prognostic meta-analysis was used to test the suitability and stability of the FRG signature. Drug sensitivity analyses were performed using the data collected from Cell Miner™.

**Results:** *PROM2, AURKA, STEAP3, CD44, ULK2, MAP1LC3A, ATP6V1G2,* and *STAT3* are among the eight genes in the FRG prognostic signature, all of which were highly expressed in stage 1 NB, except *AURKA*. Furthermore, the high-risk group, which was stratified by signature, had a lower overall survival rate than the low-risk group. GSEA revealed that high-risk groups have more biological processes related to ferroptosis.

**Conclusion:** Ferroptosis-related genes are expressed differently between stages 1 and 4 NB. The FRG signature successfully stratified NB patients into two risk groups and can accurately predict the overall survival in NB. In addition, we found that the gene *AURKA* might have the potential to be a prognostic marker in NB.

## 1 Introduction

Neuroblastoma (NB) is widely regarded as the most common solid extracranial tumor in childhood, with varying clinical manifestations and disease courses depending on tumor biology (Matthay et al., 2016). It has high morbidity in children, accounting for about 10% of pediatric cancers, and contributes significantly to pediatric cancer mortality with survival rates of less than 40% (Maris et al., 2007; Cohn et al., 2009; Matthay et al., 2016; Padovan-Merhar et al., 2016). Although scientific progress has improved the effectiveness of a variety of new treatments, such as allogeneic hematopoietic stem cell transplantation (Kanold et al., 2008), it remains at a relatively low level or requires additional research (DuBois et al., 2021; Furman et al., 2022; Heitzeneder et al., 2022).

Ferroptosis is a distinct mode of cell death caused by iron-dependent phospholipid peroxidation and governed by a number of cellular metabolic events. Since the 1950s, Harry Eagle has observed that a lack of the amino acid cyst(e)ine can cause cell death, which is similar to ferroptosis (Eagle, 1955). Nonetheless, in many ways, the field of ferroptosis is still in its infancy. Recent evidence suggests that ferroptosis may play physiological roles in tumor suppression and immunity (Jiang et al., 2021). Other studies have linked ferroptosis to NB, for example, lacking ferritin heavy chain, Erastin, or RSL3 could induce ferroptosis cell death in NB N2A cells (Lu et al., 2021), but it is unclear whether some genes that regulate ferroptosis, such as TP53 (Leu et al., 2020) and BRD4 (Sui et al., 2019), are associated with NB patient prognosis.

In this study, we identified the differentially expressed ferroptosis-related genes (FRGs) by comparing stage 1 and stage 4 NB to see if they are associated with NB prognosis. In addition, two microarrays were used in this study, and functional enrichment was used to identify additional mechanisms.

Our FRG prognostic signatures included eight differentially expressed FRGs associated with the overall survival. It performed well in predicting the prognosis of NB patients. We used the Connectivity Map database (CMAP, https://portals.broadinstitute.org/cmap/) (Lamb et al., 2006) to obtain drug target information, and data from the CellMiner database (https://discover.nci.nih.gov/cellminer/) (Shankavaram et al., 2009) were filtered to perform drug sensitivity analysis, with the goal of providing molecular strategies for the clinical diagnosis and treatment of NB.

## 2 Materials and methods

### 2.1 NB dataset processing

Agilent microarray GSE49710 datasets (*n* = 498) were downloaded from the Gene Expression Omnibus (GEO) database, and Agilent microarray E-MTAB-8248 datasets (*n* = 223) were downloaded from the ArrayExpress database. The GSE49710 and E-MTAB-8248 microarray datasets were used as discovery and validation cohorts, respectively. Patients with insufficient clinical data or in stages 2, 3, and 4 S were excluded. Table 1 shows the clinical characteristics of patients in the two cohorts.

**TABLE 1:** Clinical characteristics of the two cohorts.

	Training cohort	Validation cohort	*P*
**No. of patients**	498	223	
**Gender**			
Male	287 (57.6%)	−	
Female	211 (42.4%)	−	
**Age**			
<18 m	300 (60.2%)	103 (46.2%)	0.001
≥18 m	198 (39.8%)	120 (53.8%)	
**Mycn**			
Non-amplified	401 (80.5%)	176 (78.9%)	0.608
Amplified	92 (18.5%)	46 (20.6%)	
NA	5 (1.0%)	1 (0.4%)	
**Inss stage**			
1	121 (24.3%)	29 (13.0%)	0.013
2	78 (15.7%)	39 (17.5%)	
3	63 (12.7%)	36 (16.1%)	
4	183 (36.7%)	89 (39.9%)	
4S	53 (10.6%)	30 (13.5%)	
**Os status**			
Alive	393 (78.9%)	181 (81.2%)	0.553
Dead	105 (21.1%)	42 (18.8%)	
**Survival time (mean)**	2185.42	2225.95	0.719

### 2.2 Extraction of differentially expressed ferroptosis-related genes

FerrDb is the first manually curated resource for ferroptosis regulators and markers released in December 2019 (Zhou and Bao, 2020), from which we obtained 259 gene sets associated with ferroptosis. Differential expression analyses were performed in both training and validation cohorts using the “limma” package (Ritchie et al., 2015) and the R (version 4.1.0) software, respectively. Genes with false discovery rates (FDRs) < 0.05 and |log2FoldChange| > 0.8 were extracted as differentially expressed genes.

### 2.3 Construction of the ferroptosis-related prognostic signatures

Univariate Cox analysis of overall survival (OS) was performed to screen FRGs with prognostic values in the training cohort. *p* ≤ 0.05 was considered statistically significant. To avoid overfitting, we used the Cox proportional hazards model survival analysis with the least absolute shrinkage and selection operator (LASSO) penalty (Tibshirani, 1997). In addition, to derive a risk score for each patient, we constructed the ferroptosis-related prognostic signature by weighting the Cox regression coefficients for each gene. We classified the patients as high or low risk according to the median value in both cohorts. As for the ROC curve, we used the “survivalROC” package to evaluate the predictive ability of our established prognostic model. We assigned survival time to S time, survival status to Status, and took the risk score as a marker, and used the product-limit method to calculate the predictive ability of 3, 5, and years, respectively. For the value of AUC, it also reflects the predictive ability of our model, and the closer it is to 1, the better the ability is. In the nomogram, we considered the influence of age factors and used the calculated mortality rate as a marker. Similarly, AUC reflects the predictive ability of the Nomo model.

### 2.4 Prognostic meta-analysis of the ferroptosis-related gene signature and the gene *AURKA*

The FRG signature and the gene *AURKA* were meta-analyzed separately using the “meta” R package, including four NB datasets from the GEO (GSE62564 and GSE49710), ArrayExpress (E-MTAB-8248), and UCSC Xena (TARGET-NBL). Heterogeneity among the datasets was assessed by using the Chi2 and the I2 statistics. *p* ≤ 0.05 was considered statistically significant.

### 2.5 Function annotation and gene set enrichment analysis

The functional annotations of genes were performed using the Gene Ontology (GO) and the Kyoto Encyclopedia of Genes and Genomes (KEGG) databases. The R package “clusterProfiler” was used to analyze differentially expressed FRGs associated with OS (Yu et al., 2012). *p* values were adjusted using the BH method. In addition, the infiltrating score of 28 immune cells and the activity of 13 immune-related pathways were determined by using single-sample gene set enrichment analysis (ssGSEA) in the “GSVA” R package (Bindea et al., 2013; Hänzelmann et al., 2013).

### 2.6 Statistical analysis

The “limma” R package was used to conduct statistical analyses on differentially expressed genes. To determine the independent predictor of OS, univariate and multivariate Cox survival regression analyses were performed. In this study, all statistical analyses were conducted with the R software version 4.1.0. If not specified, *p <* 0.05 was considered statistically significant.

### 2.7 Drug sensitivity analysis of 27 differentially expressed and survival-related ferroptosis-related genes

After sorting according to log FC differential multiples, the top 500 upregulated genes and the top 500 downregulated genes were uploaded to perform the query function in the CMAP database and obtain drug target information. The Cell Miner^TM^ (Shankavaram et al., 2009; Reinhold et al., 2012) database was used to download the processed drug sensitivity data (Version: 2021.1, database: 2.6, https://discover.nci.nih.gov/cellminer/home.do) and the RNA SEQ file and compound activity. The R packages “impute,” “limma,” “ggplot2,” and “ggpubr” were used to process the data and visualize the results. Only drugs certified and clinically tested by Food and Drug Administration (FDA) are screened out, and the k-nearest neighbors in the space of genes were used to impute the missing expression values. The data of 27 differentially expressed FRGs related to prognosis were extracted. Among the gene–drug pairs with *p* < 0.001, the top 16 gene–drug pairs with the highest correlation coefficient were selected for mapping.

## 3 Results

The flow chart for this study is shown in Supplementary Figure S1, which includes 498 patients from the GEO datasets and 223 patients from the ArrayExpress database. Table 1 lists the clinical characteristics of each cohort.

### 3.1 Identification of prognostic ferroptosis-related DEGs in the training cohort

**FIGURE 1:**
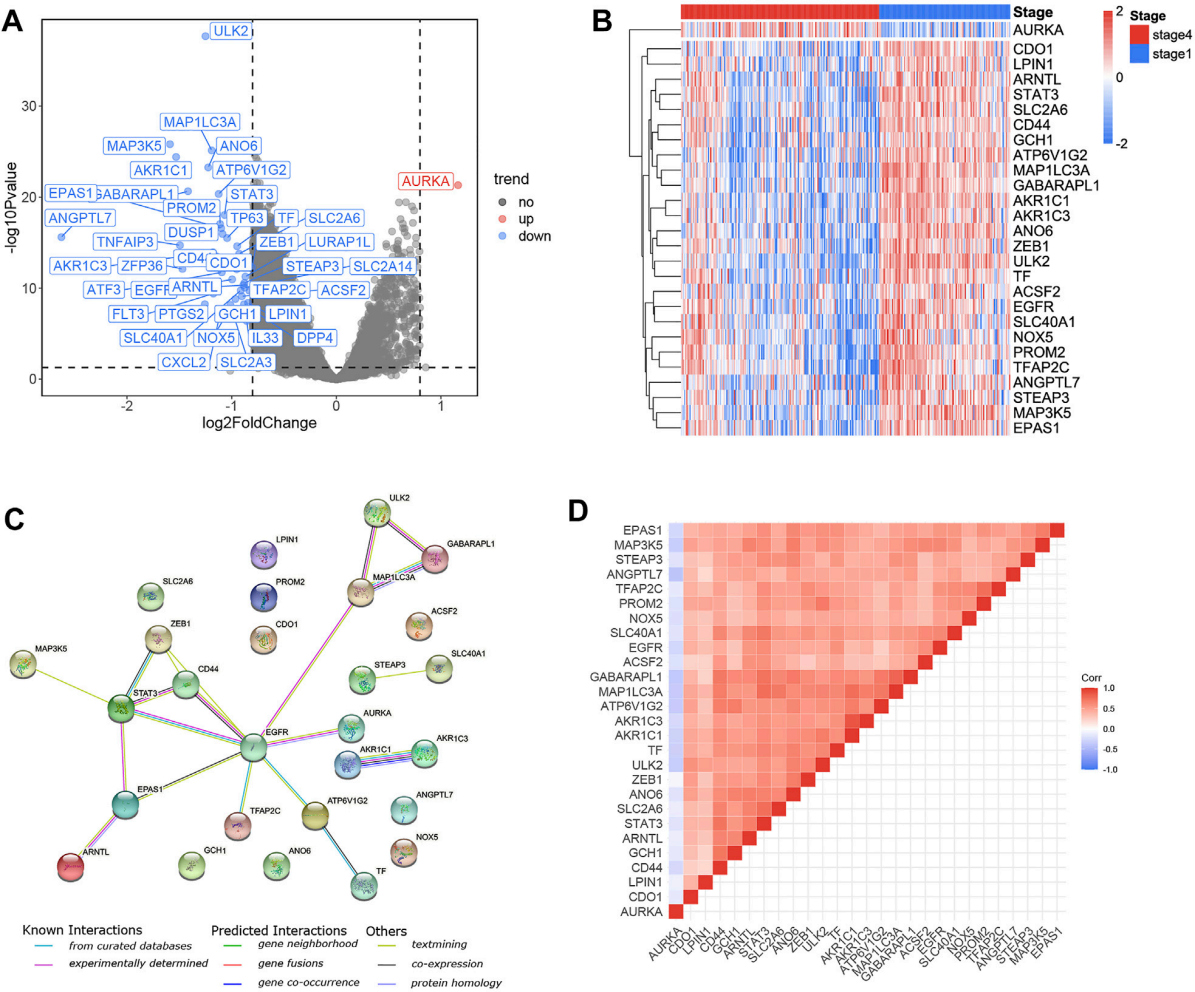
Identification of the candidate ferroptosis-related genes in the training cohort. **(A)** Volcano plot shows the differentially expressed 40 FRGs in the training cohort. **(B)** 26 genes were upregulated in stage 1 NB samples and one gene was upregulated in stage 4 NB samples. **(C)** The PPI network downloaded from the STRING database indicated the interactions among the candidate genes. The correlation heatmap of candidate genes and the correlation coefficients are represented by different colors. **(D)** The correlation heatmap shows the expression values of the identified 27 ARGs associated with OS in the training cohort.

A total of 40 FRGs were found to be differentially expressed between stage 1 and stage 4 tumor tissues (Figure 1A), and 27 FRGs were found to be significantly (*p* < 0.05) associated with OS across the entire training cohort in univariate Cox proportional model survival analyses (Supplementary Figure S2A). The majority of them (26/27) were significantly increased in stage 1 NB samples, whereas only one was significantly increased in stage 4 NB samples (Figure 1B). The PPI network indicated that EGFR and *STAT3* were the hub genes (Figure 1C). The correlation values among the 27 genes were indicated in the correlation heatmap (Figure 1D).

### 3.2 Construction and validation of FRG prognostic signatures

To eliminate false positives, the 27 FRGs that were associated with OS were subjected to LASSO Cox survival analysis (Supplementary Figure S2B, C). An eight-gene signature was identified based on the optimal value of λ (Table 2). The Kaplan–Meier curves showed the survival analyses of every gene in this signature (Supplementary Figure S3).

**TABLE 2:** FRGs in the prognostic signature.

Gene symbol	Official full name	Ensemble id	Log2FC	FDR
*PROM2*	prominin 2	ENSG00000155066	−1.11	1.85419E-16
*AURKA*	aurora kinase A	ENSG00000087586	1.16	2.57275E-20
*STEAP3*	*STEAP3* metalloreductase	ENSG00000115107	−0.88	1.64307E-10
*CD44*	*CD44* molecule (Indian blood group)	ENSG00000026508	−1.14	3.42624E-13
ULK2	unc-51 like autophagy	ENSG00000083290	−1.25	1.00798E-34
	activating kinase 2			
*MAP1LC3A*	Microtubule-associated protein 1	ENSG00000101460	−1.19	9.30898E-24
	light chain 3 alpha			
*ATP6V1G2*	ATPase H+ transporting	ENSG00000213760	−1.12	1.9092E-19
	V1 subunit G2			
*STAT3*	signal transducer and activator of transcription 3	ENSG00000168610	−1.07	2.31044E-17

**FIGURE 2:**
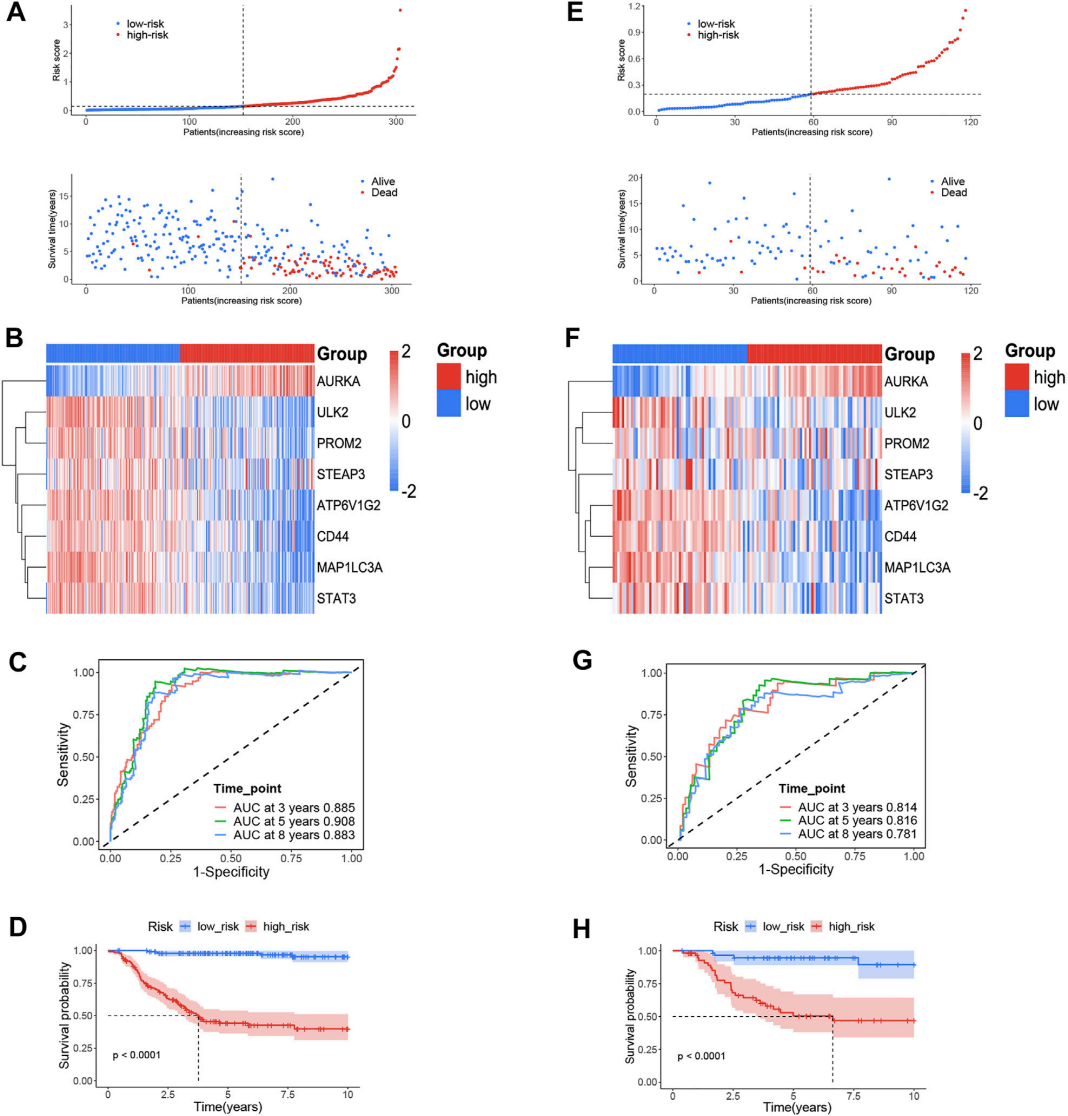
The ferroptosis-related genes (FRGs) prognostic signature for NB. **(A)** The distribution and median value of the risk scores and survival status of patients in the training cohort. **(B)** Heatmap of the FRG expression pattern in the training cohort. **(C)** Time-dependent ROC curves verified the prognostic value of the FRG signature in the training cohort. **(D)** Kaplan–Meier curves for the OS of patients in the high-and low-risk group in the training cohort. **(E)** The distribution and median value of the risk scores and survival status of patients in the validation cohort. **(F)** Heatmap of the FRG expression pattern in the validation cohort. **(G)** Time-dependent ROC curves verified the prognostic value of the FRG signature in the validation cohort. **(H)** Kaplan–Meier curves for the OS of patients in the high- and low-risk group in the validation cohort.

The risk scores were calculated for each patient as follows: risk score = e ^(– 0.339 * *ULK2*^—^0.290 *^ ^*MAP1LC3A*^ ^+ 0.581 *^ ^*AURKA*^—^0.107 *^ ^*ATP6V1G2*^—^0.168 *^ ^*STAT3*^—^0.044 * *PROM2*^—^0.075 * *CD44*^ ^+ 0.258 * *STEAP3*)^. We used the median value as the cut-off value, and the training cohort was divided into high-risk group (*n* = 152) and low-risk group (*n* = 152), which represent the increased and decreased ferroptosis, respectively. Figure 2A depicts the risk distribution, survival status, and gene expression pattern in the training cohort. According to the scatter plot, the high-risk group had a higher probability of death earlier than the low-risk group in the training cohort because the majority of patients in the low-risk group survived the 15-year follow-up, whereas the majority of patients in the high-risk group died (Figure 2A). According to the heatmap, whether in the training cohort or in the validation cohort, seven FRGs, including *PROM2, STEAP3, CD44, ULK2, MAP1LC3A, ATP6V1G2,* and *STAT3*, were highly expressed in stage 1 NB, while only *AURKA* was highly expressed in stage 4 NB (Figures 2B,F). We discovered that the FRG signature has good predictive performance in predicting OS in the training cohort using the time-dependent ROC curves, with the area under the curve (AUC) reaching 0.885 at 3 years, 0.908 at 5 years, and 0.883 at 8 years (Figure 2C). The Kaplan–Meier curve revealed that patients in the high-risk group had a significantly lower OS than those in the low-risk group (Figure 2D). To validate the model’s prognostic significance, the same risk score formula was used to test the FRG signature in the validation cohort (*n* = 223). The validation cohort as a whole was divided into two groups using the same cut-off value as the training cohort. Figure 2E depicts the risk distribution, survival status, and gene expression pattern in the validation cohort. Time-dependent ROC curves were used to assess the predictive performance of the risk score model for OS, and the AUC values at 3, 5, and 8 years were 0.814, 0.816, and 0.781, respectively (Figure 2G). The Kaplan–Meier curve consistently showed similar results to the training cohort, indicating that the high-risk group had a clearly worse OS than the low-risk group (Figure 2H).

### 3.3 The expression status of *AURKA* in different subgroups

**FIGURE 3:**
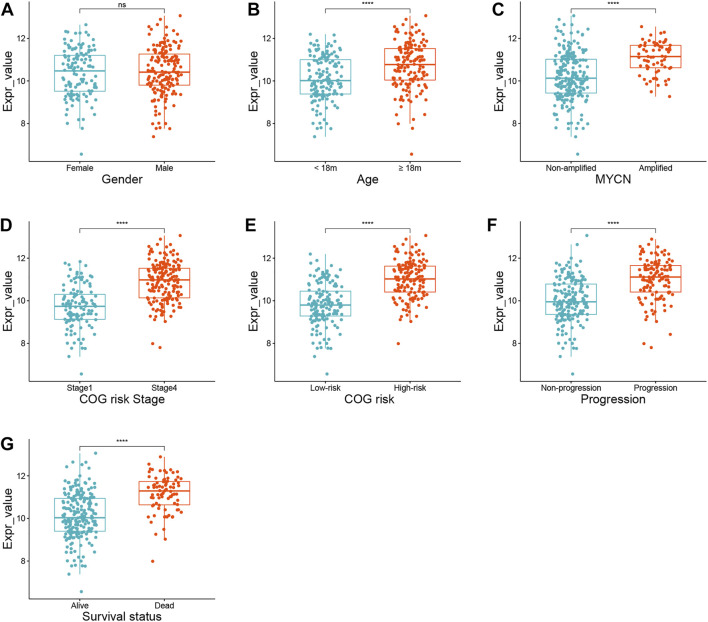
The expression status of *AURKA* in different subgroups. **(A)** Gender. **(B)** Age. **(C)** MYCN amplification status. **(D)** INSS stage. **(E)** COG risk status. **(F)** Progression. **(G)** Survival status.

An interesting phenomenon was discovered in the heatmap of the training and validation cohorts: only one gene, *AURKA*, had a high expression level in stage 4 tumors or high-risk groups. In the training cohort, we compared the expression status of *AURKA* in seven subgroups, including gender, age, MYCN amplification status, INSS stage, COG risk status, progression, and survival status. Figures 3B–G show that *AURKA* expression was higher in patients aged 18 months (*p <* 0.001), MYCN amplified (*p <* 0.001), stage 4 NB (*p <* 0.001), COG high risk (*p <* 0.001), progression (*p <* 0.001), and dead status (*p <* 0.001) when compared to the corresponding groups. However, no significant difference was found between the male and female groups (Figure 3A). Supplementary Figure S4 shows a comparison of *AURKA* expression status in the validation cohort.

### 3.4 The prognostic value of ferroptosis-related gene signatures

**FIGURE 4:**
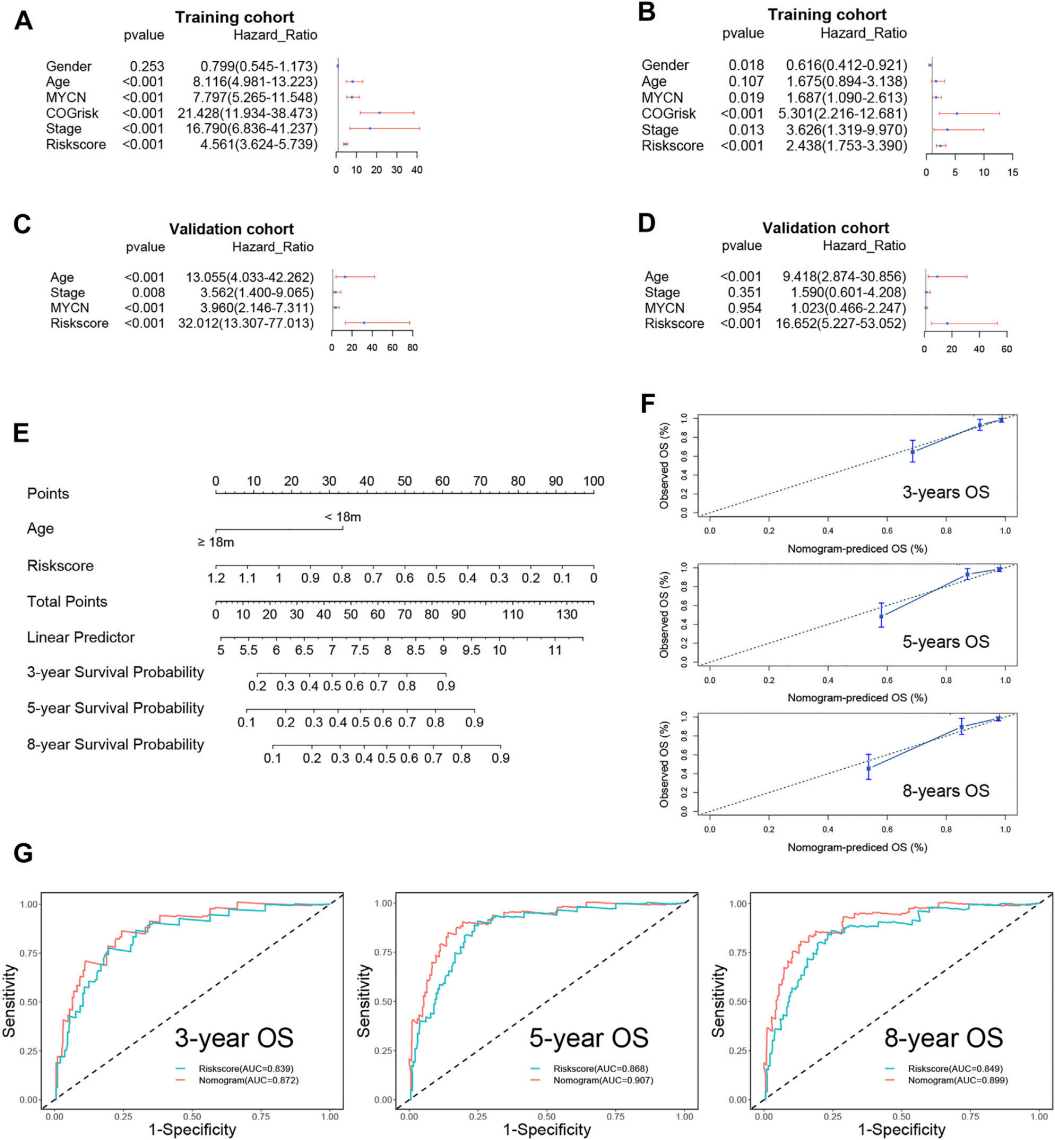
Univariate and multivariate survival analyses regarding OS in the training cohort and the validation cohort. **(A)** Univariate Cox regression analyses in the training cohort. **(B)** Univariate Cox regression analyses in the validation cohort. **(C)** Multivariate Cox regression analyses in the training cohort. **(D)** Multivariate Cox regression analyses in the validation cohort. **(E)** The nomogram for predicting overall survival in the validation cohort. **(F)** The calibration curves of 3-, 5-, and 8-years for the nomogram. **(G)** The 3-, 5,- and 8-year ROC curve analyses for the nomogram.

The risk score and other clinical risk factors in the entire training cohort were subjected to univariate and multivariate Cox regression analyses (Figure 4A). In the univariate survival analysis, the risk score was significantly associated with OS in both the training [hazard ratio (HR) = 4.561; 95% CI: 3.624–5.739; *p* < 0.001] and validation cohorts [hazard ratio (HR) = 32.012; 95%CI:13.307–77.013; *p* < 0.001] (Figures 4A,C). After evaluating gender (female vs male), age status (<18 vs ≥18 months), MYCN amplification (non-amplified vs amplified), Children’s Oncology Group (COG) risk status (low risk vs high risk), and International Neuroblastoma Staging System (INSS) stage (INSS 1 vs INSS 4), it was determined that the FRG signature plays an independent prognostic role (Training cohort: HR = 2.438, 95% CI = 1.753–3.390, *p* < 0.001; validation cohort: HR = 16.652, 95% CI = 5.227–53.052, *p* < 0.001; Figures 4B,D). Given that the COG risk classification already incorporates age, MYCN amplification status, and INSS stage factors, we created a nomogram (Figure 4E) incorporating only age and the FRG signature risk score to further investigate the role of age in predicting the OS in NB patients in the validation cohort, as age showed significant differences in univariate and multivariate Cox regression analyses. Age ≥18 months was assigned a value of 0, while age <18 months was assigned a value of 33.6. Furthermore, a risk score of 1.2 was defined as 0 points, while a risk score of 0 was defined as 100 points. Risk scores ranging from 1.2 to 0 correspond to points ranging from 0 to 100, and can be calculated using the following formula: points = (1.2 - risk score) * (100/1.2).

The C-index of the nomogram was 0.85 (95% CI: 0.80–0.91), indicating that our signature is highly accurate. Additionally, the3-, 5-, and 8-year calibration curves demonstrated that the predicted OS was quite consistent with the observed OS (Figure 4F). The ROC curve revealed that the nomogram’s AUC at 3, 5, and 8 years was greater than the risk score’s AUC at 3, 5, and 8 years (Figure 4G).

### 3.5 The prognostic role of ferroptosis-related gene signatures

Stratification survival analyses were used to determine the FRG signature’s predictive performance in clinical subgroups within the training cohort. The entire training cohort was divided into 11 subgroups according to gender, age, MYCN not amplified status, INSS stage, COG risk status, and progression.

**FIGURE 5:**
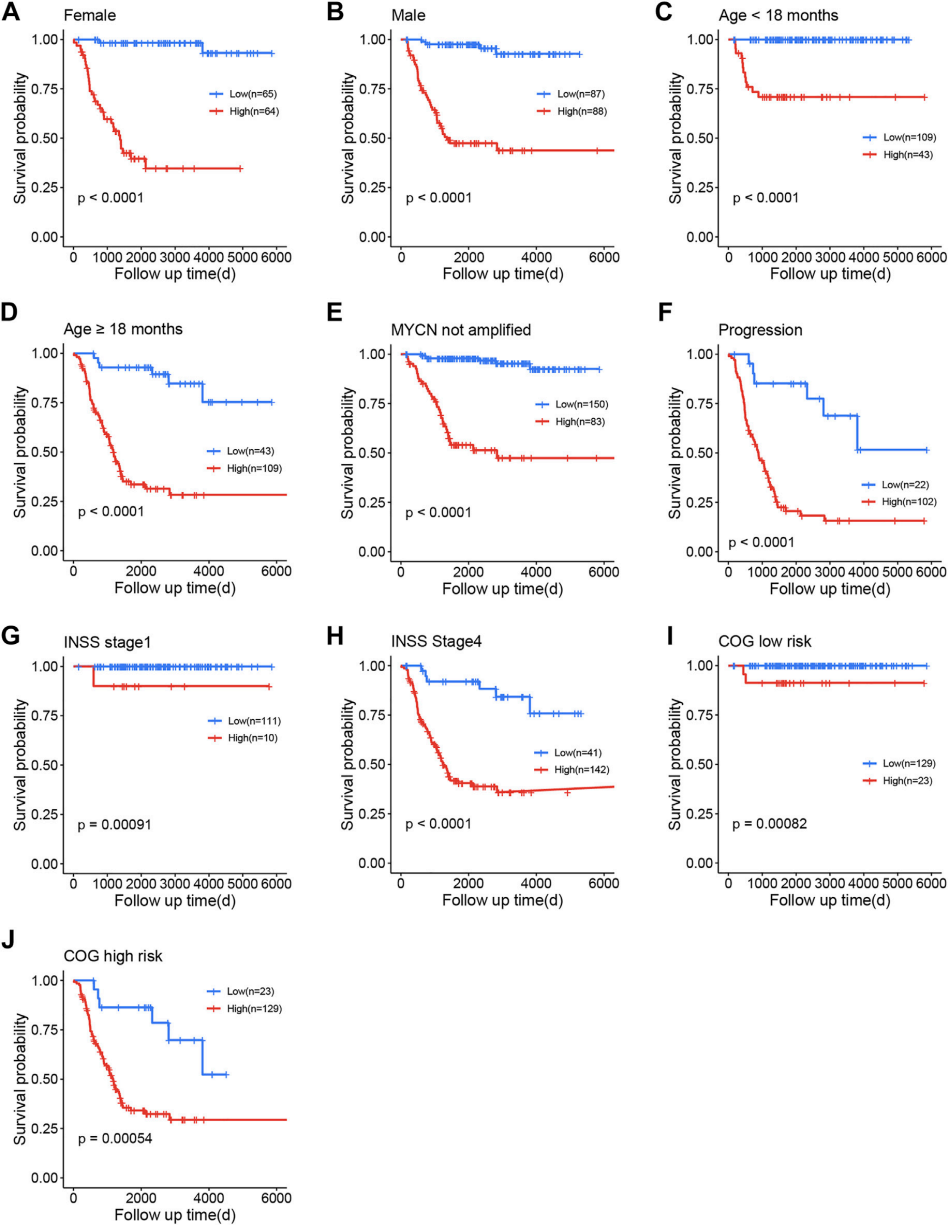
Survival analysis of the training cohort. **(A)** Female. **(B)** Male. **(C)** Age <18 months. **(D)** Age ≥18 months. **(E)** MYCN not amplified. **(F)** Progression. **(G)** International Neuroblastoma Staging System (INSS) stage 1. **(H)** INSS stage 4. **(I)** Children’s Oncology Group (COG) low risk. **(J)** COG high risk.

Patients were divided into high- and low-risk subgroups in each subgroup using the same cut-off value as in the training cohort. A significant difference was demonstrated in the OS between the high- and low-risk groups (Figures 5A–J).

### 3.6 Meta-analysis of ferroptosis-related gene signatures and the gene AURKA

The prognostic meta-analysis was used to assess the comprehensive predictive value of the FRG prognostic signature in multiple cohorts. The results revealed that the FRG prognostic signature was a significant predictor of cancer prognosis in NB (HR = 8.75, 95% CI: 1.44–53.19, *p* = 0.02, Supplementary Figure S5). In addition, by meta-analysis, we validated the gene *AURKA* as a potential prognostic biomarker for NB (HR = 4.41, 95% CI: 1.19–16.36, *p* = 0.03, Supplementary Figure S6).

### 3.7 Functional analyses for the training and validation cohorts

**FIGURE 6:**
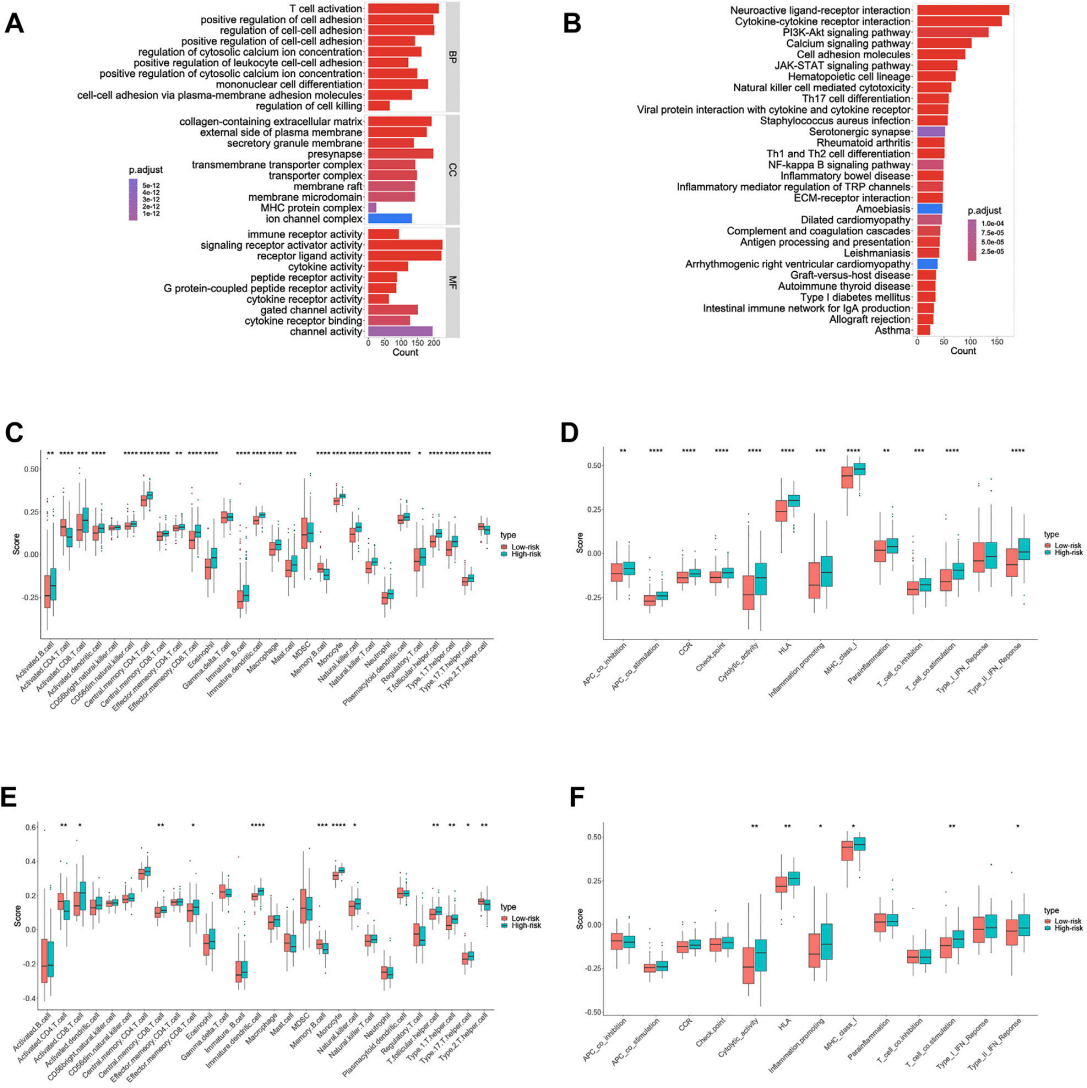
Results of GO and KEGG analyses and comparison of the ssGSEA scores between the high- and low-risk group in the training cohort. **(A)** Results of GO enrichment in the training cohort. **(B)** Results of the KEGG pathway in the training cohort. **(C)** Scores of 16 immune cells in the training cohort. **(D)** Scores of 13 immune-related functions in the training cohort. **(E)** Scores of 16 immune cells in the validation cohort. **(F)** Scores of 13 immune-related functions in the validation cohort.

To further investigate the relationship between biological function and pathway and risk score, we included differentially expressed and survival-related FRGs in our GO enrichment (Figure 6A) and KEGG pathway analyses (Figure 6B) in the high- and low-risk groups. As expected, the differentially expressed FRGs were significantly more abundant in iron-related signaling pathways, such as the PI3K-Akt signaling pathway. Surprisingly (Yi et al., 2020), the FRGs were also significantly enriched in a variety of immune-related biological processes, including T-cell activation, positive regulation of leukocyte cell–cell adhesion, and mononuclear cell differentiation. Following that, we used the ssGSEA to determine the degree of enrichment of immune cell subpopulation-related pathways. Numerous immune-related cells were found to be significantly different between the low- and high-risk groups in the training cohort, including activated CD4 T cells, eosinophils, macrophages, and neutrophils (Figure 6C). Clearly, numerous antigen-presenting functions, such as APC costimulation, HLA, cytolytic activity, and MHC class I expression, appear to be distinct between the two groups (Figure 6D).It is to be noted that the KEGG-enriched cytokine–cytokine receptor interaction had a higher score in the training cohort’s high-risk group. Additionally, the high-risk group scored higher on immature dendritic cells, monocyte, cytolytic activity, and T-cell costimulatory activity (Figures 6C,D), which was validated in the validation group (Figures 6E,F).

### 3.8 Exploration of drugs that have a therapeutic effect on NB based on the FRG signature

We screened the Cmap datasets for the significant interaction nodes in order to identify potential drugs that modulate prognosis-related genes therapeutically. A key node is one that interacts with at least two genes associated with prognosis and has a hypergeometric *p*-value less than 0.05. Table 3 summarizes the 20 most significant drugs.

**TABLE 3:** Drugs in the Cmap datasets.

Rank	Cmap name	Mean	N	Enrichment	*p*-value
1	harmol	0.744	4	0.965	<0.00001
2	vorinostat	−0.708	12	−0.821	<0.00001
3	trichostatin A	−0.641	182	−0.731	<0.00001
4	thioridazine	−0.371	20	−0.514	<0.00001
5	LY-294002	−0.419	61	−0.484	<0.00001
6	tanespimycin	−0.265	62	−0.371	<0.00001
7	trifluoperazine	−0.468	16	−0.541	0.00002
8	wortmannin	−0.460	18	−0.522	0.00002
9	Prestwick-675	0.638	4	0.920	0.00004
10	fluphenazine	−0.385	18	−0.509	0.00008
11	15-delta prostaglandin J2	−0.387	15	−0.539	0.00014
12	isoxicam	0.398	5	0.848	0.00018
13	niclosamide	−0.635	5	−0.832	0.00032
14	alvespimycin	−0.250	12	−0.573	0.00032
15	nortriptyline	−0.720	4	−0.882	0.00046
16	meteneprost	0.556	4	0.864	0.00046
17	quinostatin	−0.790	2	−0.984	0.00054
18	tetracycline	0.372	5	0.812	0.00058
19	sirolimus	−0.300	44	−0.290	0.00102
20	loperamide	−0.470	6	−0.711	0.00135

### 3.9 Sensitivity analysis of drugs for NB

**FIGURE 7:**
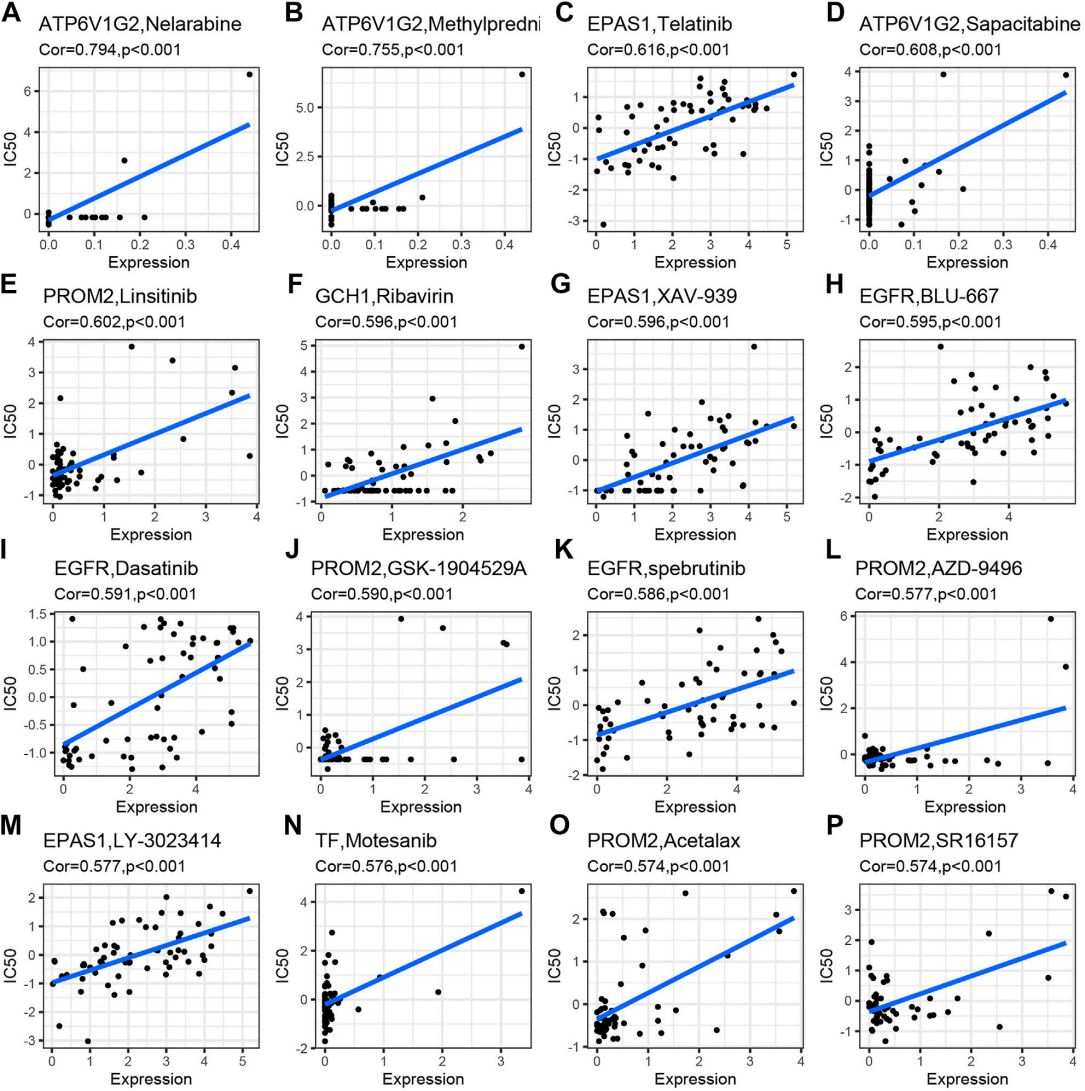
Drug sensitivity analysis of differentially expressed and survival-related FRGs. Drug sensitivities of Nelarabine **(A)**, Methylprednisolone **(B)**, and Sapacitabine **(D)** were positively related to *ATP6V1G2* expression. Drug sensitivities of Telatinib **(C)**, XAV-939 **(G),** and LY-3023414 **(M)** were positively related to EPAS1 expression. Drug sensitivities of Linsitinib**(E)**, GSK-1904529A **(J)**, AZD-9496 **(L)**, Acetalax **(O),** and SR16157 **(P)** were positively related to *PROM2* expression. Drug sensitivity of Ribavirin **(F)** was positively related to GCH1 expression. Drug sensitivities of BLU-667 **(H)**, Dasatinib **(I),** and spebrutinib **(K)** were positively related to EGFR expression. Drug sensitivity of Motesanib **(N)** was positively related to TF expression.

The correlation analysis was performed to investigate the potential relationship between drug sensitivity in different human cell lines and the 27 differentially expressed and survival-related FRGs. The results are shown in Figure 7 from high to low in terms of the absolute value of the correlation coefficient, indicating that *ATP6V1G2* expression was associated with drug sensitivity to nelarabine, methylprednisolone, and sapacitabine (Figures 6A,B,D). The expression of one hub gene, *EFGR*, correlated positively with the drug sensitivity of BLU-667, Dasatinib, and Spebrutinib (Figures 6H,I,K). There was a positive relationship between the expression of another hub gene, EPAS1, and the drug sensitivity of Telatinib, XAV-939, and LY-3023414 (Figures 6C,G,M). The relationship between *PROM2* expression and drug sensitivity of Linsitinib, GSK-1904529A, AZD-9496, Acetalax, and SR16157 was also found to be positive (Figures 6E,J, L, O, P). Furthermore, the correlation between *GCH1* expression and Ribavirin drug sensitivity and the correlation between *TF* expression and Motesanib drug sensitivity were positive (Figure 6N).

## 4 Discussion

In light of recent advances in the treatment of NB, the International NB Risk Group Staging System (INRGSS) has been adopted prospectively for the treatment assignment and definition of COG clinical trial eligibility (Irwin et al., 2021). Furthermore, researchers have identified several prognostic signatures or markers that may serve as critical reference points for clinicians during the therapeutic regimen (Formicola et al., 2016; Suo et al., 2018; Zhang et al., 2018; Gao et al., 2020; Meng et al., 2020; Zhong et al., 2021), but only a small proportion of patients benefit from them. According to our knowledge, this is the first study to attempt to establish a correlation between ferroptosis and prognostic FRGs in NB using RNA-seq data.

Some researchers have recently reported that ferroptosis has an inhibitory effect on NB. One study, for example, found that high-risk NB could be eradicated by the nano-targeted induction of dual ferroptotic mechanisms (Hassannia et al., 2018). They discovered that by creating a specific type of nanoparticle, they could mimic the canonical ferroptosis-inducing pathway, which is a powerful strategy for treating high-risk NB. Furthermore, another study found that knocking out ferroportin accelerates ferroptosis induced by erastin in NB cells (Geng et al., 2018). However, studies have discovered that ferroptosis has tumor-protective properties. For example, one study found that by incorporating iron into mitochondria, FtMt could inhibit erastin-induced LIP elevation and reduce ROS content in SH-SY5Y cells, thus protecting the NB SH-SY5Y cells from ferroptosis injury (Wang et al., 2016). In another study, ferrostatin-1 was found to be capable of inhibiting ferroptosis in dopaminergic NB SH-SY5Y cells exposed to rotenone-induced oxidative stress (Kabiraj et al., 2015).

When the gene characteristics of stage 1 and stage 4 tumors were compared and screened, eight genes were found to have the best prognostic value and were screened out of gene sets from FerrDb to put into our FRG signature. We also performed survival analyses for all the genes in the signature (Supplementary Figure S5). According to the analyses, we found that all the genes except *STEAP3* (*p* > 0.05) have strong predictive ability. Furthermore, prognostic meta-analysis confirmed that the FRG signature was an independent prognostic predictor in multiple cohorts, although the survival difference failed to reach a significant level in the TARGET-NBL cohort. An interesting phenomenon was discovered in these genes: only one gene, *AURKA*, had a high expression level in stage 4 tumors and high-risk groups, whereas others were highly expressed in stage 1 or low-risk groups. Ramani et al.(2015) discovered that *AURKA* is a potentially valuable diagnostic indicator of survival in NB using immunohistochemistry. It has been reported that it is a direct negative regulator of necrosome activation and that high levels of mRNA expression of this gene are associated with poor survival (Xie et al., 2017). Previous microarray analyses showed that MYCN-amplified NBs had higher levels of *AURKA* mRNA than nonamplified NBs (Berwanger et al., 2002) and this was confirmed in another study (Otto et al., 2009), which showed that *AURKA* was not only required for the growth of MYCN-amplified NB cells but also for cells lacking amplified MYCN, which is consistent with the study of Roeschert et al. (2021). In addition, it has been reported that changing the conformation of the *AURKA* activation loop with small molecules can effectively disrupt the *AURKA*/N-myc interaction in NB cancer cells (Boi et al., 2021), and using selinexor and the *AURKA* inhibitor alisertib to synergistically increase the cytotoxicity of p53-mediated high-risk NB has potential therapeutic benefits (Nguyen et al., 2022). As a result, we believe that *AURKA* may open up new avenues for biomarkers used in the prognosis of NB.

*PROM2*, *STEAP3*, *CD44*, *ULK2*, *MAP1LC3A*, *ATP6V1G2*, and *STAT3* are different from *AURKA*. *PROM2* is involved in iron metabolism and may contribute to ferroptosis resistance by promoting the formation of multivesicular bodies containing iron-laden ferriti, resulting in a decrease in the intracellular iron concentration (Brown et al., 2019). Furthermore, the overexpression of *CD44* suppressed ferroptosis in cancer cells in an OTUB1-dependent manner (Liu et al., 2019), and (Monteleone et al. (2021) demonstrated that inhibiting protein kinase C, which can modulate *CD44* expression, is a strategy to sensitize NB stem cells to etoposide by stimulating ferroptosis. A high *STEAP3* expression causes lipid peroxidation of the cellular membrane (Howie et al., 2019), implying that it may inhibit NB cell growth in this way. When the activity of *ULK2*, an autophagy-related protein, was increased, it was found to be capable of promoting the normal dissolution of stress granules (Wang et al., 2019). *MAP1LC3A* is an autophagy-related gene that encodes microtubule-associated protein 1 light chain 3 alpha (LC3) (Miao et al., 2021). *ATP6V1G2* encodes the Na+/H+ antiporter, and its high expression improves ion control in epithelial cells (Albecker et al., 2021). *STAT3* is a signal transducer and transcription activator, and CTSB (cathepsin B) mediated by *STAT3* is required for ferroptosis (Gao et al., 2018). Studies have shown that by regulating the STAT pathway, tumor immunosuppression in the NB can be alleviated through the targeted elimination of bone marrow-derived suppressor cells (Xu et al., 2021). These could be the underlying mechanisms of the genes’ high expression in stage 1 NB and low expression in stage 4 NB.

According to our drug sensitivity analyses, *ATP6V1G2* is associated with nelarabine, a type of drug targeting T-cell antigens (Salvaris and Fedele, 2021), methylprednisolone, which can improve the opsoclonus myoclonus syndrome associated with NB (Zhu et al., 2021), and sapacitabine, a nucleoside analogue inducing DNA strand breaks (Liu et al., 2012), which has been reported to play a role in the treatment of advanced solid (Denlinger et al., 2021) tumor. *EPAS1* is sensitive to the multi-tyrosine kinase inhibitor telatinib (van et al., 2018), the Wnt signaling pathway antagonist XAV-939 (Wang et al., 2021), and the dual PI3K/mTOR inhibitor LY3023414 (Chen et al., 2021). However, there have been few studies in recent years on the relationship between telatinib, XAV-939, or LY3023414 and NB. *PROM2* is sensitive to insulin-like growth factor I receptor inhibitor linsitinib (Fernando et al., 2021), insulin-like growth factor 1 inhibitor GSK1904529A (Zeng et al., 2021), the estrogen receptors alpha antagonist AZD9496 (Cani et al., 2021), and the dual-acting estrogen action inhibitor SR16157 (Rausch et al., 2011). Among them, linsitinib has been reported to have potent antitumor activity in diffuse midline glioma when combined with modified chimeric antigen receptor T-cells (de Billy et al., 2021); GSK1904529A has also been found to inhibit glioma tumor growth, induce apoptosis, and inhibit migration (Zhou et al., 2015), and AZD9496; and SR16157 are potential drugs for treating breast cancer that are present in the early stages of clinical research (Zhang et al., 2021) (Rausch et al., 2011). *GCH1* is sensitive to ribavirin, a synthetic nucleoside analogue with broad antiviral activity (Jurković et al., 2021). *EGFR* was sensitive to the small-molecule rearranged inhibitor BLU667 (Subbiah et al., 2018), the tyrosine kinase inhibitor dasatinib (Vitali et al., 2009), and the Bruton’s tyrosine kinase (BTK) inhibitor spebrutinib during transfection (Kaliamurthi et al., 2021). BLU-667 has been shown to induce tumor regression in cancer models with RET mutations and fusions (Subbiah et al., 2018), dasatinib has been shown to reduce NB growth as early as 2009 (Vitali et al., 2009), and spebrutinib has antitumor activity in large B-cell lymphoma (Tanaka et al., 2020). Motesanib, an antiangiogenic receptor tyrosine kinase inhibitor, is effective against *TF* (Torok et al., 2017). Sensitivity analyses indicate that these drugs may have therapeutic potential for NB, as they have a strong positive correlation with the expression of genes that are highly expressed in stage 1 tumors.

The relationship between tumor susceptibility and ferroptosis has been a hot topic in recent years, but the potential mechanism of tumor immunity and ferroptosis remains unknown. GO analyses were performed on the basis of differentially expressed FRGs between the high- and low-risk groups, and we discovered that these genes were enriched in many immune-related biological processes and pathways, implying a link between NB immunity and ferroptosis. There are also significant differences in the process of antigen presentation between the two groups. One possible mechanism is that ferroptotic cells communicate with immune cells *via* a set of signals that include lipid mediators, which attract antigen-presenting cells (APCs) and other immune cells near the ferroptotically dying cells (Friedmann Angeli et al., 2019).

In general, this study demonstrated the ferroptosis-related gene signatures in NB associated with the prognosis and proposed the possibility of the *AURKA* gene as a prognostic marker in NB, which is consistent with many preclinical studies (Berwanger et al., 2002; Otto et al., 2009; Ramani et al., 2015; Xie et al., 2017; Boi et al., 2021; Roeschert et al., 2021; Nguyen et al., 2022). We call for more attention to *AURKA*, expecting to open up a new way to treat NB.

## 5 Conclusion

Ferroptosis-related genes are expressed differently between stages 1 and 4 NB. The FRG signature successfully stratified NB patients into two risk groups and can accurately predict the overall survival in NB. In addition, we found that the gene *AURKA* might have the potential to be a prognostic marker in NB.

## Data Availability

The datasets presented in this study can be found in online repositories. The names of the repository/repositories and accession number(s) can be found in the article/Supplementary Material.
