# Supplementary figures and images for: Ferroptosis-related gene signatures in neuroblastoma associated with prognosis
Source: Front Cell Dev Biol. 2022 Sep 6;10:871512. doi: 10.3389/fcell.2022.871512 (PMC9486025; doi:10.3389/fcell.2022.871512)

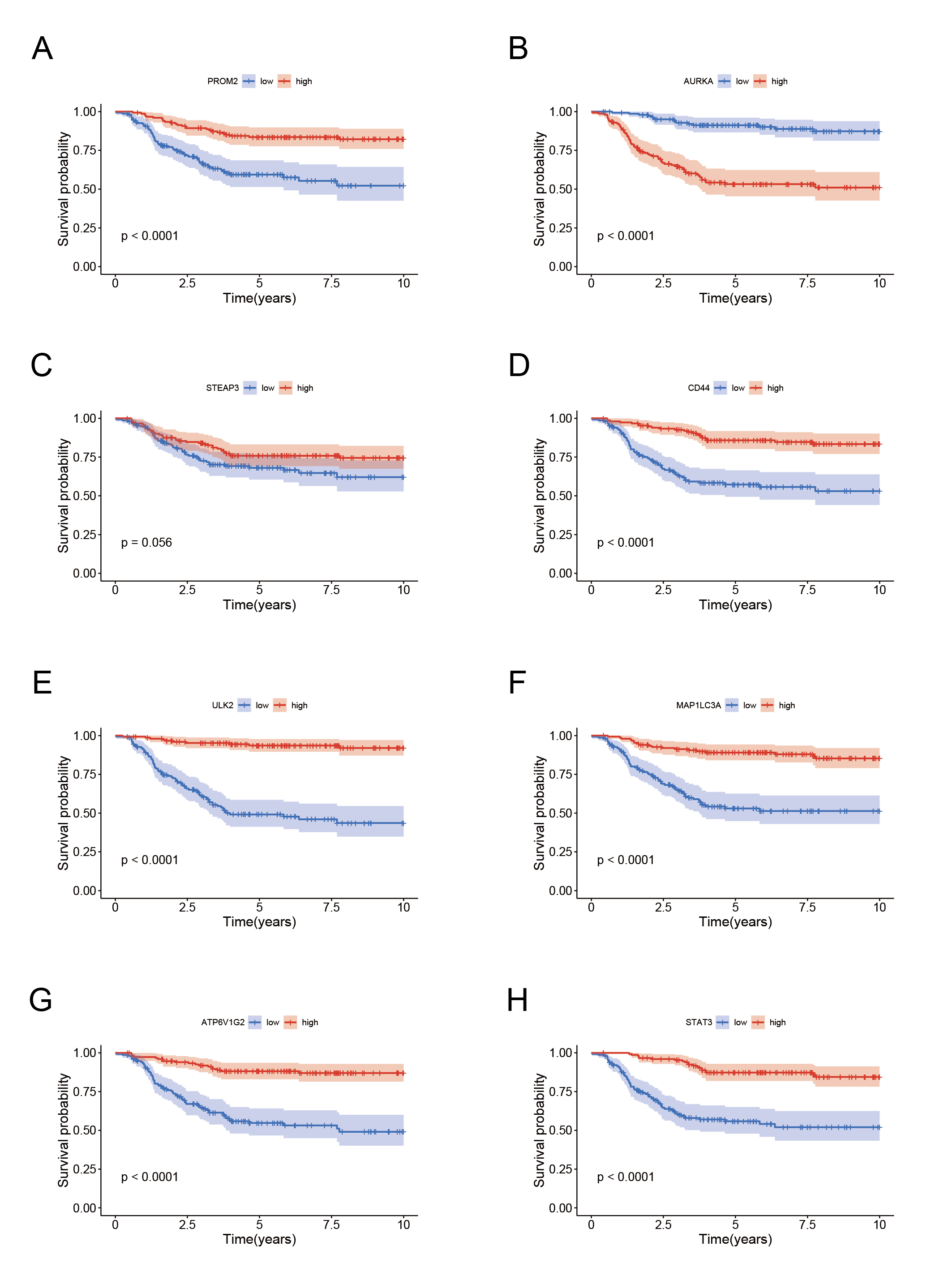

Supplement: Supplementary file 1 [file Image3.JPEG]

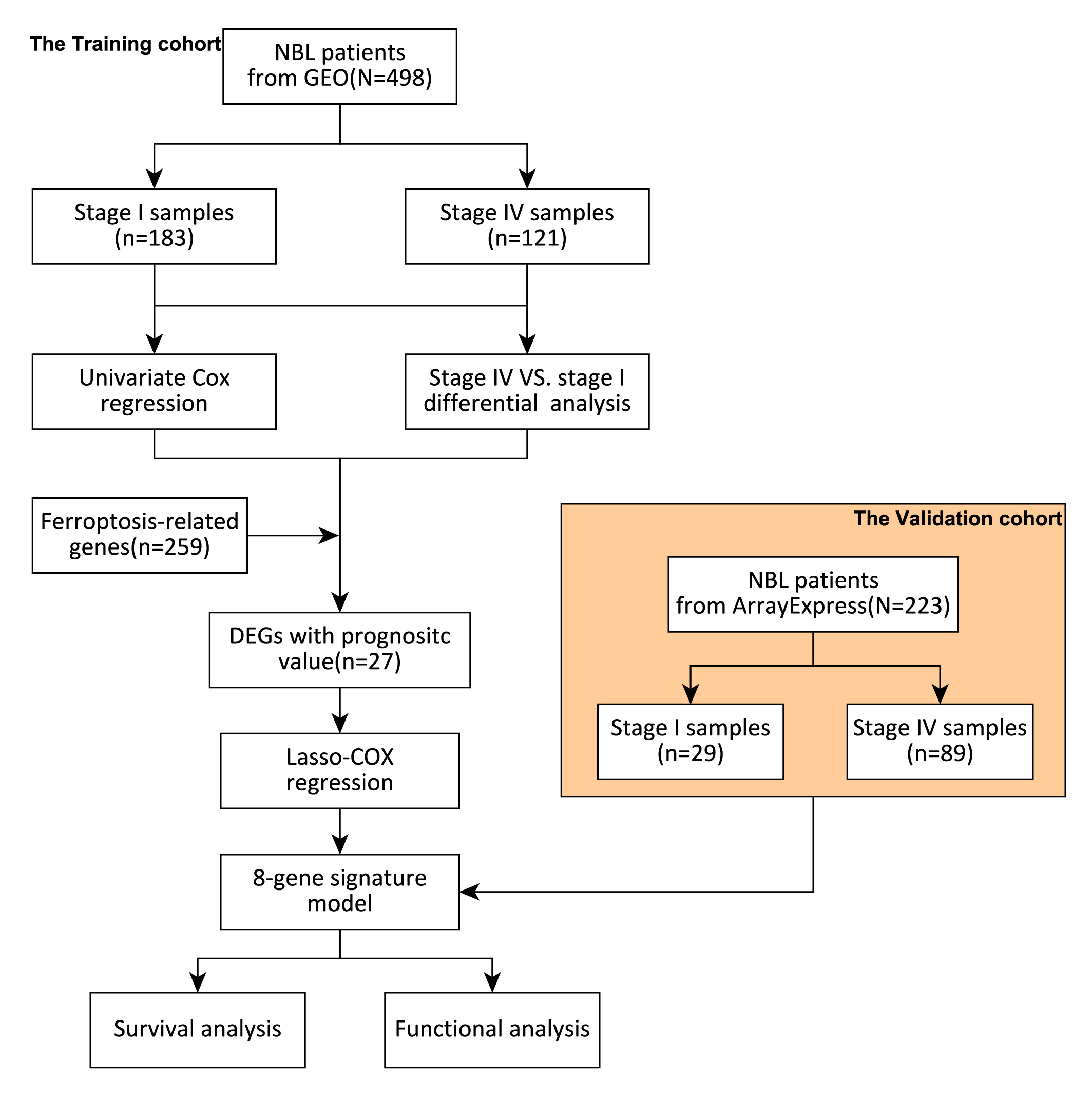

Supplement: Supplementary file 2 [file Image1.JPEG]

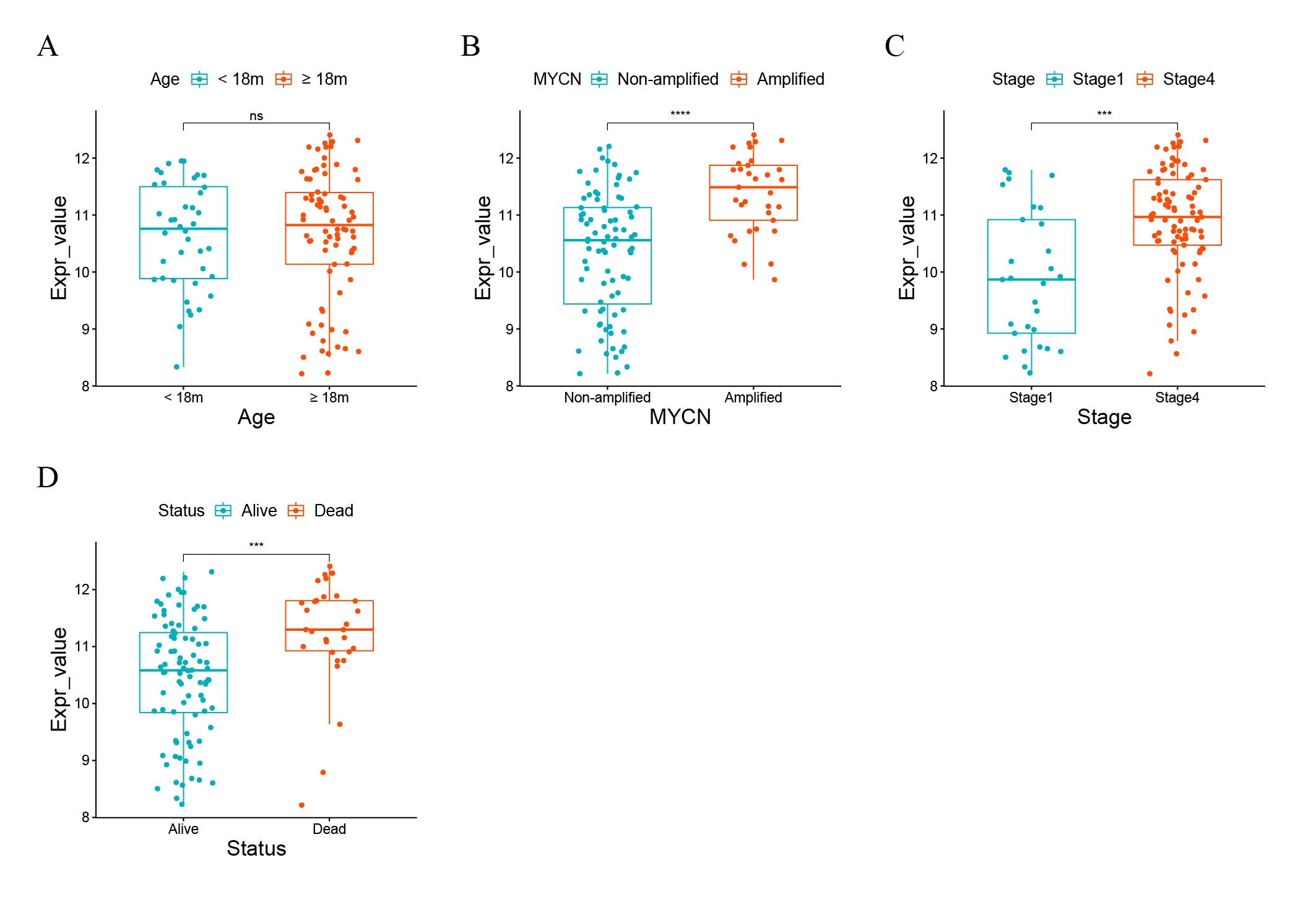

Supplement: Supplementary file 3 [file Image4.JPEG]

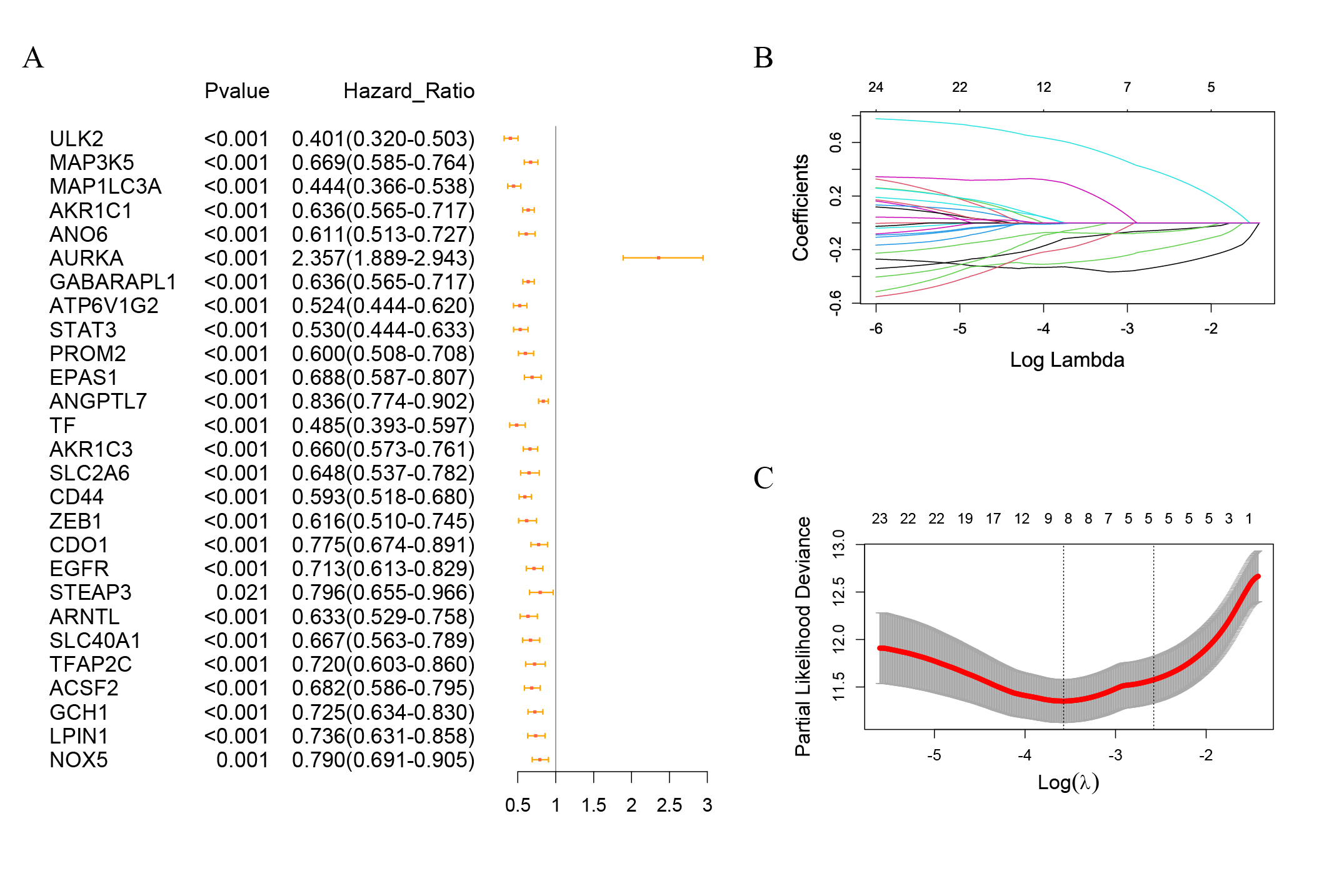

Supplement: Supplementary file 4 [file Image2.JPEG]

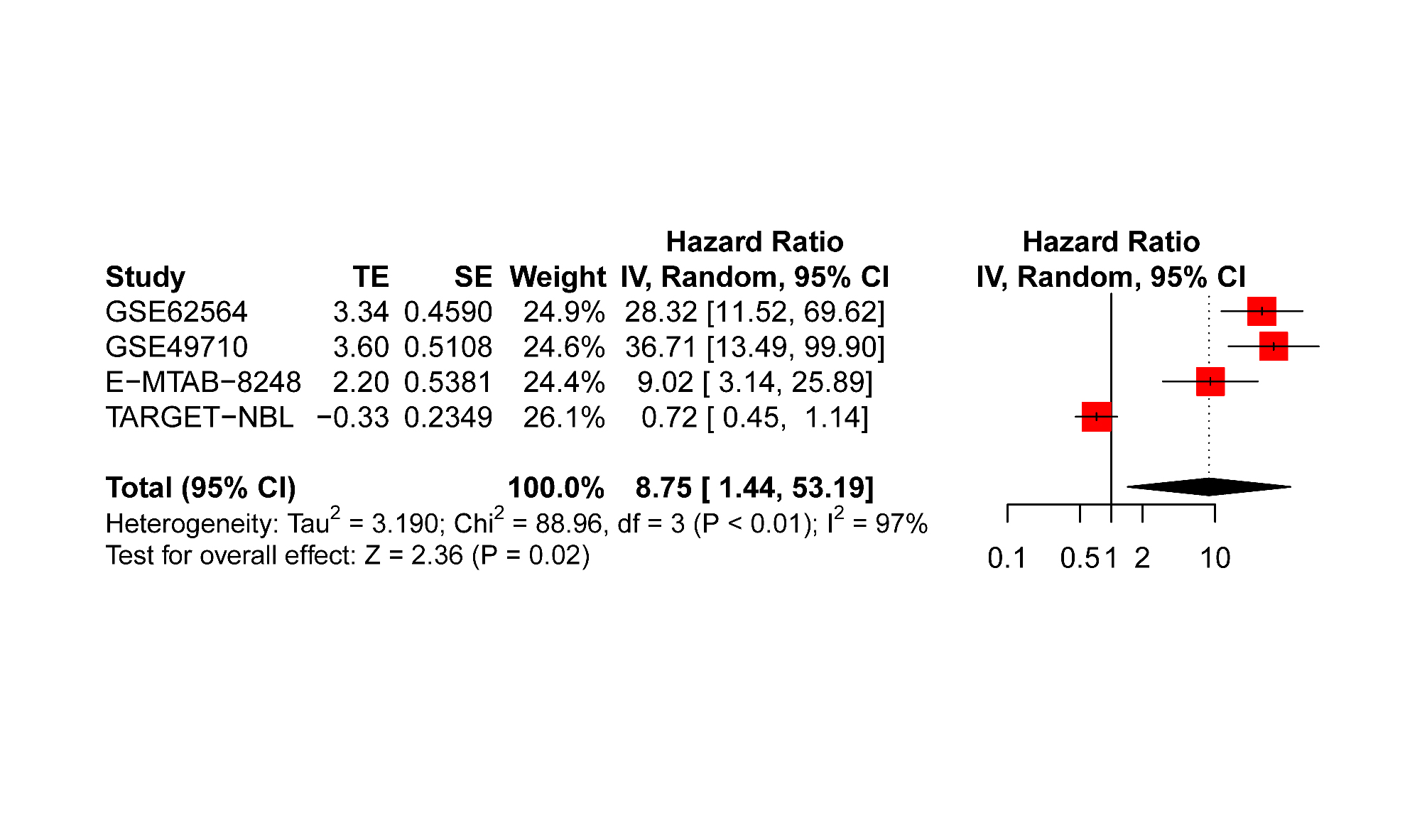

Supplement: Supplementary file 5 [file Image5.JPEG]

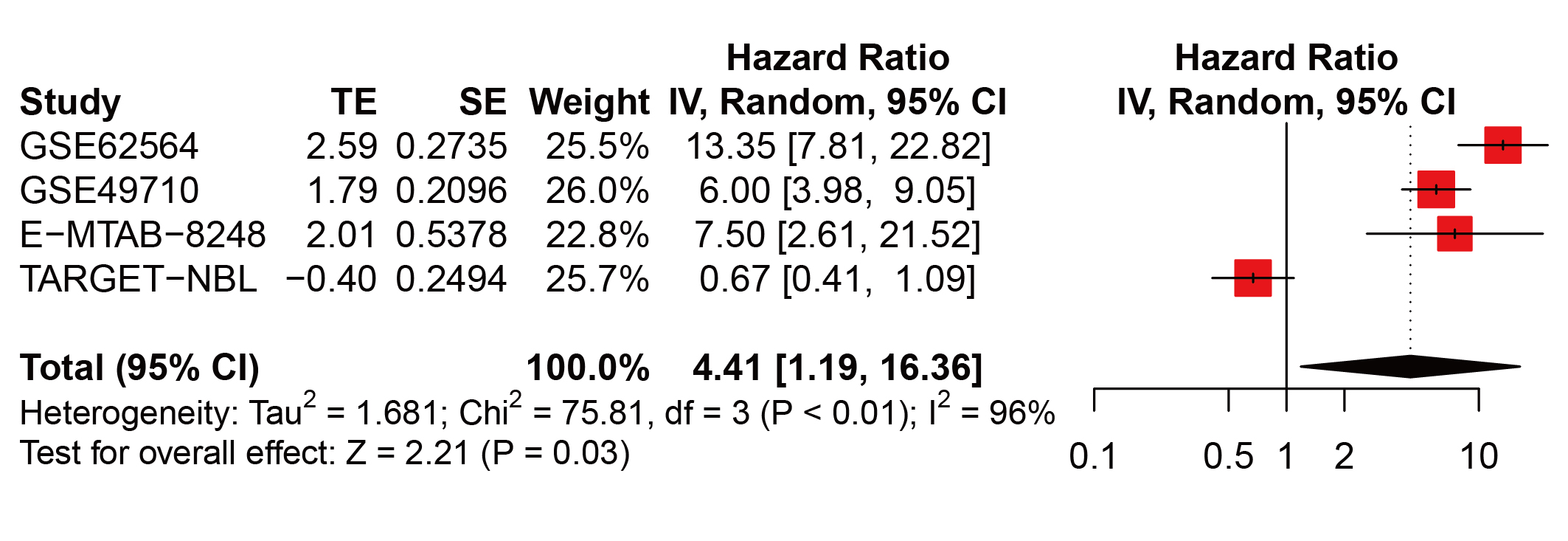

Supplement: Supplementary file 6 [file Image6.JPEG]
